# Hardware Implementation of 32-Bit High-Speed Direct Digital Frequency Synthesizer

**DOI:** 10.1155/2014/131568

**Published:** 2014-06-02

**Authors:** Salah Hasan Ibrahim, Sawal Hamid Md. Ali, Md. Shabiul Islam

**Affiliations:** ^1^Department of Electronics, College of Engineering, Diyala University, Baqubah, Diyala 32001, Iraq; ^2^Department of Electrical, Electronics & System Engineering, Faculty of Engineering, Universiti Kebangsaan Malaysia (UKM), 43600 Bangi, Malaysia; ^3^Institute of Microengineering and Nanoelectronics (IMEN), Universiti Kebangsaan Malaysia (UKM), 43600 Bangi, Malaysia

## Abstract

The design and implementation of a high-speed direct digital frequency synthesizer are presented. A modified Brent-Kung parallel adder is combined with pipelining technique to improve the speed of the system. A gated clock technique is proposed to reduce the number of registers in the phase accumulator design. The quarter wave symmetry technique is used to store only one quarter of the sine wave. The ROM lookup table (LUT) is partitioned into three 4-bit sub-ROMs based on angular decomposition technique and trigonometric identity. Exploiting the advantages of sine-cosine symmetrical attributes together with XOR logic gates, one sub-ROM block can be removed from the design. These techniques, compressed the ROM into 368 bits. The ROM compressed ratio is 534.2 : 1, with only two adders, two multipliers, and XOR-gates with high frequency resolution of 0.029 Hz. These techniques make the direct digital frequency synthesizer an attractive candidate for wireless communication applications.

## 1. Introduction

Direct digital frequency synthesis (DDFS) systems with high-speed frequency hopping function, low power consumption, high frequency resolution, and small chip area are in a great demand for DDFS applications especially in wireless communications and radar systems. DDFS systems have low noise and high frequency resolution compared with phase lock loop [1]. The DDFS output frequency (*F*_out_) is given by

(1) $$\begin{array}{c} \;F_{\text{out}}=\left(\frac{\text{FCW}}{2^{N}}\right)*F_{\text{clk}}, \end{array}$$

where *F*_clk_ is clock frequency, FCW is a frequency control word, and *N* is the phase accumulator width. Pipelining technique is defined as a technique that partitioned the given task into a number of subtasks that need to be performed in a sequence. The pipelining technique is used in [2–4] to increase the throughput of the output frequency. However, this technique also increases the power consumption and chip area. The gated clock technique was applied to the pipeline phase accumulator (PA) in [5] to reduce the number of registers without performance degradation. A parallel adder based on progression-of-states technique was combined with the pipelining technique in [6, 7], for high-speed, low power pipelined parallel PA. The pipelined accumulator with parallel adder technique was proposed in [8]. In this paper, two blocks of parallel 16-bit ripple carry adder (RCA) are designed based on the progression-of-states technique with two pipelining stages.

DDFS spectral purity depends on the number of the phase output bits used for addressing the ROM. Therefore, the higher spectral purity required, the bigger ROM size needed. The quarter wave symmetry in [9, 10] was applied as a simple technique to reduce the ROM size, storing only quarter (0 : *π*/2) of the sine wave bits and using the two most significant bits (MSB) to generate the full sine wave (2*π*). Quarter wave symmetry together with an angular decomposition method based on trigonometric identity was used in [11–13] to compress the ROM size by partitioning the ROM into three sub-ROMs. This technique was also used in [14], where the ROM is partitioned into two ROMs, namely, coarse and fine ROMs, to obtain the quadrature outputs, sine and cosine values. The author suggested several DDFS systems implemented with different types of FPGA kit boards and compared them with an ASIC based approach.

The polynomial approximation method was used in DDFS design to optimize the spurious-free dynamic range (SFDR). This method was used by Ashrafi and Adhami in [15] to determine the upper bound of the SFDR using piecewise polynomial interpolation. Fourier series was used to establish a linear relationship between the coefficients of the interpolating polynomials and the frequency spectrum. This method can be used in any kind of DDFS to maximize the SFDR.

De Caro et al. proposed a dual-slope technique in [16] to optimize the piecewise linear approximation for the phase to sine mapping. This technique improves the spectral purity and ROM size. The stored values of the two quarters (*π*/4) sine and cosine sub-ROMs, with 3 MSB bits and mapper, are required to accomplish the quarter phase to sine mapping and produce quadrature output from the DDFS. In [17], De Caro et al. proposed another DDFS architecture which is based on piecewise linear approximation technique with nonuniform segment length to the input of three groups of multiplexer. This technique can maximize the SFDR and reduce the size of the coefficients ROM.

This paper presents a high-speed DDFS system with pipelined PA based on modified parallel BK adder and gated clock technique. The ROM was resized by applying the quarter-wave symmetry technique in one quarter of the sine wave, and an angular decomposition technique based on trigonometric identity has been applied to compress the quarter ROM LUT. Based on these techniques, the quarter ROM LUT was partitioned into three sub-ROMs (*A*, *B*, and *C*). The proposed architecture improves the speed of the DDFS and reduces the size of the ROMs.

## 2. The Gated Clock and Parallel Pipelining Technique for Phase Accumulator

The modified parallel BK adder based on the progression-of-states technique combined with gated clock technique was used in the proposed design of the PA. The frequency resolution (*F*_Res_) of DDFS is determined by the clock frequency (*F*_clk_) and the number of *N* input bit of the PA as depicted by

(2) $$\begin{array}{c} F_{\text{Res}}=\frac{F_{\text{clk}}}{2^{N}}. \end{array}$$

**Figure 1:**
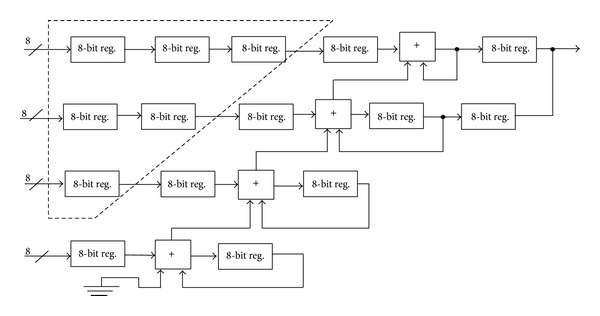
The conventional 32-bit pipelining phase accumulator.

For high frequency resolution, it is preferable to design a PA with large FCW bits input. However, a large ROM size is required to implement all the 2^*N*^ bits of phase accumulator output. Due to this reason, a part of the MSB phase output is used to address the phase to amplitude converter or ROM lookup table while maintaining high frequency resolution. The pipeline technique was used to increase the throughput of the accumulator, and this throughput will double with the number of pipeline stages, as shown in Figure 1.

**Figure 2:**
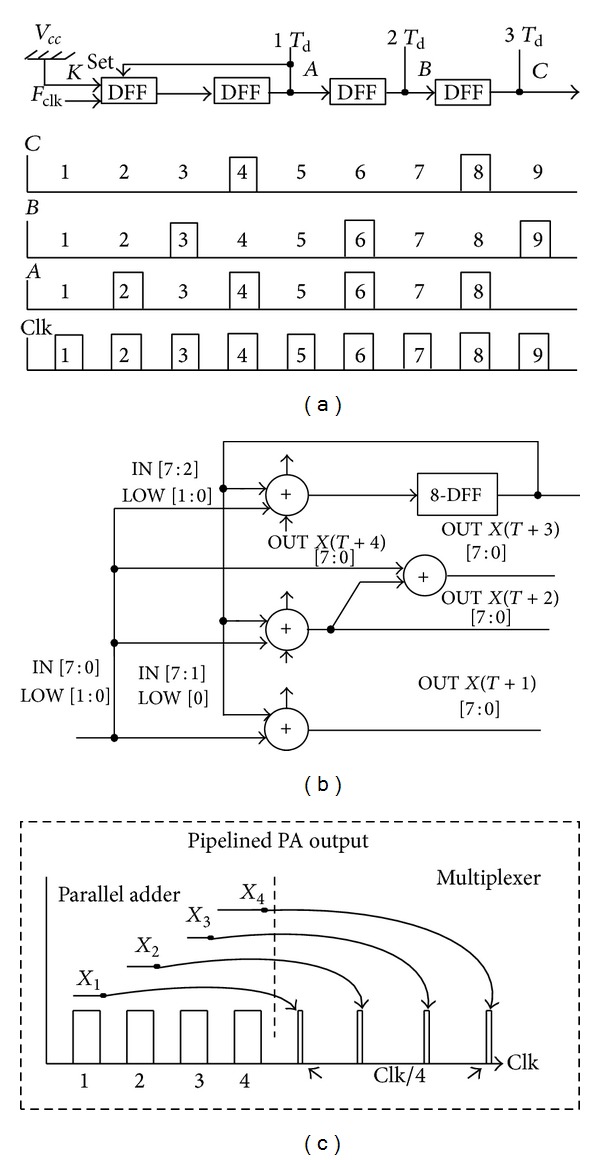
(a) The gated clock technique, (b) parallel adder based on progression-of-states technique, and (c) pipelined PA output.

The number of registers increases with the number of pipeline stages, which leads to high power consumption. Therefore, in this design, a gated clock technique was used to reduce the number of preskewing registers while preserving high-speed operation. In this technique, D flip-flops (DFFs) were used to connect each row of the pipeline stages with FCW input. These registers are clocked by the pipelined pulses with one clock cycle based on the shifted clock pulses as shown in Figure 2(a). Considering that the phase accumulator input bits are *N*, the PA was partitioned into *L* stages with B DFFs in each stage. The number of the DFFs, *K* for preskewing registers, is given by

(3) $$\begin{array}{c} K=\frac{\left(N*\left(L+1\right)\right)}{2}. \end{array}$$

By applying the gated clock technique on the proposed design, the number of DFFs is given by

(4) $$\begin{array}{c} K=N+L. \end{array}$$

As a result, with the gated clock technique, the numbers of preskewing registers have been reduced from 80 to 36 corresponding to 53.7% reduction.

Figure 2(b) shows the operation of the parallel adder. Four adders calculate the holding constant of 8-bit input word within four clock cycles. Holding the FCW for four clock cycles limits the update rate of the frequency input word but does not cause any glitch, because the four adders will steadily increase the accumulator output four times. Assume that FCW is *N* and the parallel adder output is *X* at the *T*th clock time. The parallel adder's outputs are given as follows:

(5) X(T+1)=N+X(T),X(T+2)=X(T+1)+X(T)=N+X(T)+X(T)=N+2X(T),X(T+3)=X(T+2)+X(T)=N+3  X(T),X(T+4)=X(T+3)+X(T)=N+4X(T).

The result of the fourth adder is set as a feedback to the second input for all the four adders. To generate the second output [*X*(*T* + 2)] and fourth output [*X*(*T* + 4)], the *N* bit is shifted up by removing one and two bits of the FCW input and replacing them with the one and two bits from the lower pipelining stage, respectively, before they are added. The *X*(*T* + 2) output and *X*(*T*) output are used to generate *X*(*T* + 3).

This operation made the frequency tuning word held constant for four clock cycles without causing any imperfections in the PA output. The partitioned clock cycles (*Clk*/4) make the multiplexers choose one of the results at the output of the PA to overcome the holding time on the parallel adders as illustrated in Figure 2(c).

## 3. Phase Accumulator Architecture

**Figure 3:**
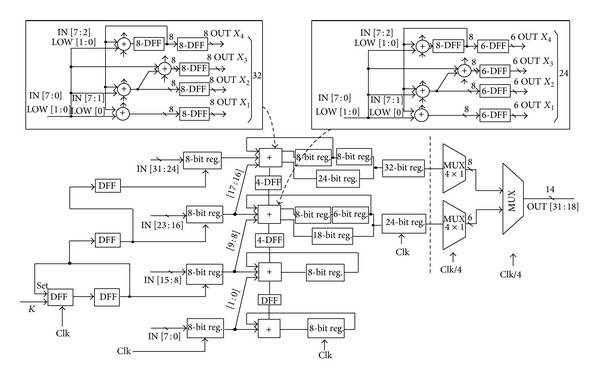
Pipelined phase accumulator with parallel adder based on modifying Bren-Kung adder and gated clocking technique.

The proposed phase accumulator architecture based on the modified parallel BK adder and the gated clock technique with pipelining stages is shown in Figure 3. The output of the PA is a truncated 14-bit value that is achieved from the 8 and 6 bits of the top and second pipelining stages, respectively.

The 32-bit FCW input is sufficient to obtain 0.029 Hz frequency resolution from the 125 MHz clock frequency of Cyclone III FPGA kit board (*F*_Res_ = 125 × 10^6^/2^32^ = 0.029 Hz).

### 3.1. Modifying Brent-Kung Adder

**Figure 4:**
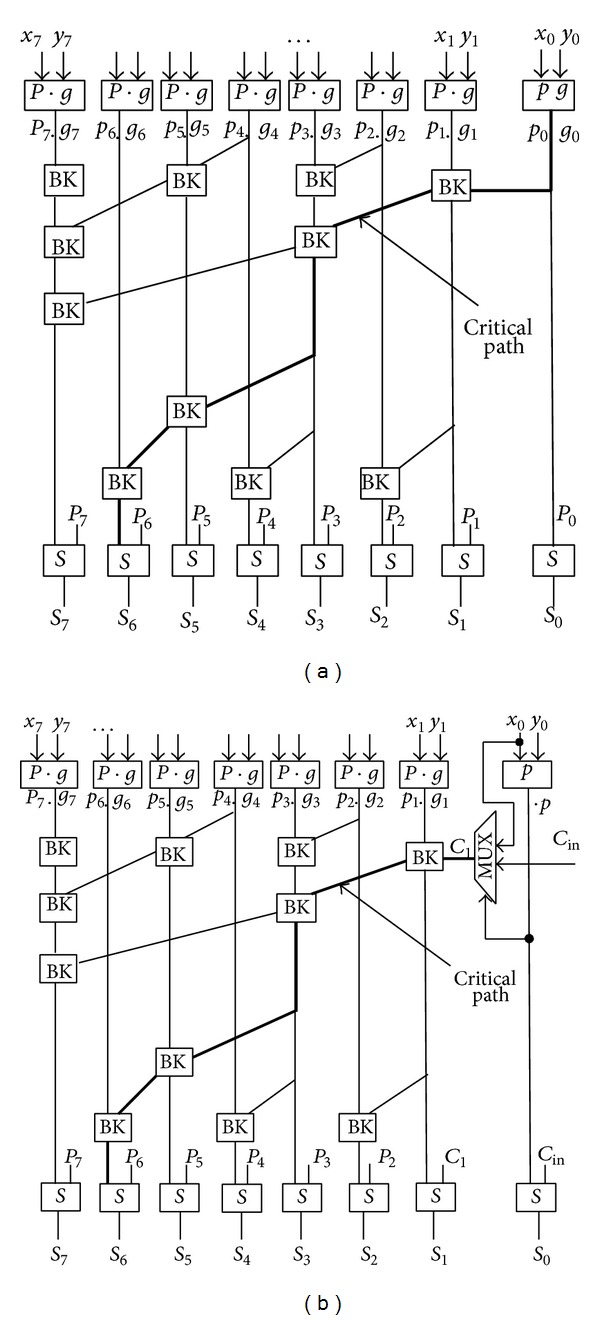
Block circuit diagram of conventional Brent-Kung adder (a) and (b) modifying Brent-Kung adder.

The BK adder is fast, and all carries are computed simultaneously through a binary tree of “BK” cells as shown in Figure 4(a).

BK cells compute the carry for two or more of full adder (FA) cells, and they are calculated as a sum of *G* and *P* cells [23, 24]. The arithmetic operation is given by

(6) $$\begin{array}{c} \text{BK}=G+P,\\ G=g^{\prime \prime }+p^{\prime \prime }\cdot g^{\prime },\\ P=p^{\prime \prime }\cdot p^{\prime }, \end{array}$$

where *g*′′*g*′, *p*′′*p*′ are higher and lower generate and propagate functions, respectively. The *p* is a propagate function (*p*_*i*_ = *x*_*i*_ ⊕ *y*_*i*_) and *g* is a generate function (*g*_*i*_ = *x*_*i*_ · *y*_*i*_).

The general prefix addition algorithm is explained by Zimmermann in [25]. By adding the carry input *C*_in_ in the prefix structure with some modifications, the prefix structure can be used in pipelining-based adder design. This approach is used in BK adder fast carry computation. However, in this paper, a modification is proposed to the BK adder so that it can be used in pipelining architecture. The proposed modification is by removing the operation of the *g*_0_, and the carry out of the first bit *C*_1_ can be achieved by a 2-1 multiplexer. The input to this multiplexer is *x*_0_ and *C*_in_ while *p*_0_ is the select input and the output is *C*_1_. The operation of the multiplexer is given by

(7) $$\begin{array}{c} C_{1}=\left(p_{0}.\;c_{\text{in}}\right)+\left(x_{0}.\;\overset{-}{p_{0}}\right), \end{array}$$

where *C*_1_ is the carry out, *p*_0_ is the propagate function, *C*_in_ is the carry input, and *x*_0_ is the first bit input. The proposed modification of the 8-bit BK adder is shown in Figure 4(b). The sum and carry out of the modified 8-bit BK adder are shown in

(8) $$\begin{array}{c} S_{0}=p_{0}⊕c_{\text{in}},\\ S_{N\cdots 1}=p_{\left((N-1)\cdots 1\right)}⊕C_{\left((N-1)\cdots 1\right)},\\ C_{\text{out}}=g_{N}+p_{N}\cdot C_{N}. \end{array}$$

#### 3.1.1. Comparison of Different Adder Architecture

An adder is a key element of the pipelining PA design, and a fast adder improves PA performance. Parallel-prefix adder tree structures such as Sklansky [26], Kogge-Stone adder [27], BK [23], and Beaumont-Smith [28] have been used in pipelining accumulator design for high-speed operation.

**Figure 5:**
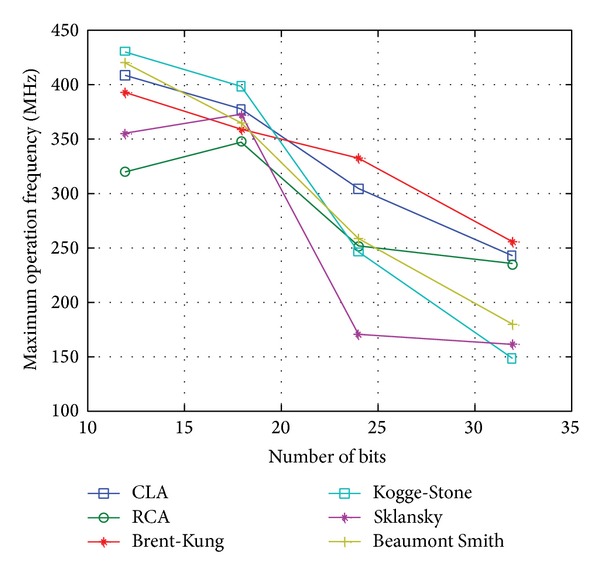
Comparison result of maximum operating frequency (*F*_*Max*⁡_) for phase accumulator design with CLA, Brent-Kung, Kogge-Stone, Sklansky, Beaumont-Smith, and RCA adders.

A comparison has been made between conventional adder and several parallel-prefix adders for 12-bit, 18-bit, 24-bit, and 32-bit operations. The PA designs were coded in Verilog HDL and verified in Cyclone III FPGA kit board. Prior to that, all the designs were simulated by using ALTERA Quartus II. The comparison result is shown in Figure 5. From the figure, it can be seen that BK adder performs relatively faster, especially for high number of bits.

## 4. ROM Lookup Table Design

ROM LUT or phase-to-amplitude converter (PAC) is a memory storage address for DDFS, which is used to convert the phase signal into an amplitude sine wave signal. High-accuracy output signal for DDFS requires a large number of LUT. The ROM size exponentially increases with increasing number of bit inputs. The designer's challenge is a tradeoff between reducing the ROM size while maintaining high performance (high resolution, high speed). Quarter-wave symmetry technique is used to resize the ROM in the proposed DDFS design to store only one quarter (0 : *π*/2) of the sine waveform and two most significant bits (MSB) from the phase accumulator are used to reconstruct the full sine wave. From these two MSBs, one of them is used to determine if the sine amplitude is increasing or decreasing and the other one is used to determine its sign.

The phase output is directly used in the first and third quarter while the inverse values of the phase output are used in the second and fourth quarters. This requires 2's complement when the phase is between (*π* : 2*π*) and is achieved by adding full adder at the output gate to accomplish the full sine wave value. To save power and achieve a smaller area design, the 1/2 LSB offset is added to the stored memory address of sub-ROMs. This offset removes the full adder component from the 2's complement of the proposed design.

The angular decomposition technique based on trigonometric identity is one of the best techniques to reduce the quarter ROM LUT size. The quarter ROM LUT was partitioned into three (*A*, *B*, and *C*), such that *A* < (*π*/2), *B* < (*π*/2)∗(1/2^*A*^), and *C* < (*π*/2)∗(1/2^*A*+*B*^), with the same approximations based on trigonometric identity.

According to the trigonometric relation, the sine wave function is given in

(9) sin(A+B+C) =sin(A+B)cos⁡C+cos⁡Acos⁡BsinC  −sinAsinBsinC,

where

(10) $$\begin{array}{c} \sin\left(A+B\right)=\sin A\cos B+\cos A\sin B. \end{array}$$

Equation (10) indicates the presence of four split sub-ROMs, two (2^*A*^ and 2^*B*^) for sin and cos⁡*A* and *B*. The calculated results of (sin-cos*A*) show that they are inversely symmetrical. Based on this, cos⁡(*A*) can be obtained by complementing the sin(*A*) values and this is achieved by connecting the sin(*A*) output and the high *V*_*cc*_ to the XOR logic gate inputs. In this way, only one addressing sub-ROM is needed for sin*A* and cos⁡*A* values. The equation for cos⁡*B* calculation is given by

(11) $$\begin{array}{c} \cos B=\cos\left(\left(\frac{\pi }{2}\times \frac{\left[0:\left(2^{B}-1\right)\right]}{2^{B}}\right)\times \left(\frac{1}{2^{B}}\right)\right). \end{array}$$

The formula shows that the cos⁡*B* values with 4-bit input are fed into the sub-ROM *B*(*B* = 4); therefore cos⁡*B* = cos⁡(0.046019) = 0.999999677≅1. Thus, the (cos⁡*B*) block ROM was also removed from the proposed design. Therefore, (10) can be reduced as given by

(12) $$\begin{array}{c} \sin\left(A+B\right)=\sin A+\cos A\sin B. \end{array}$$

With three values of *A*, *B*, and *C*, (9) may be rewritten as

(13) $$\begin{array}{c} \sin\left(A+B+C\right)=\sin A+\cos A\sin B+\cos A\sin C. \end{array}$$

**Figure 6:**
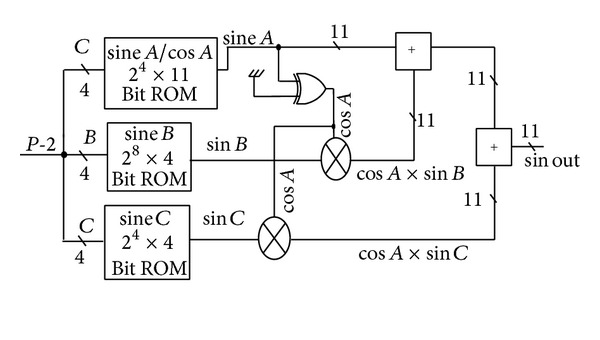
Architecture of compressed ROM LUT design.

The change in the proposed ROM LUT design after hardware reduction is shown in Figure 6.

The error that results from the approximation in (9) is (2*π*/2^14^), which is approximately 3.83 × 10^−4^. The required ROM size for 12-bit input is 2^14^ × 12 = 196608 bits. Applying the sine wave approximation based on trigonometric identity with three 4-bit ROM requires only 368 bits [(2^4^ × 11 = 176) + (2^4^ × 8 = 128) + (2^4^ × 4 = 64)] for ROM *A*, ROM *B*, and ROM *C*, respectively. The compressed ratio (196608/368) is 534.2 : 1, with only two adders, two multiplayer adders, and XOR gate as additional hardware equipment.

**Figure 7:**
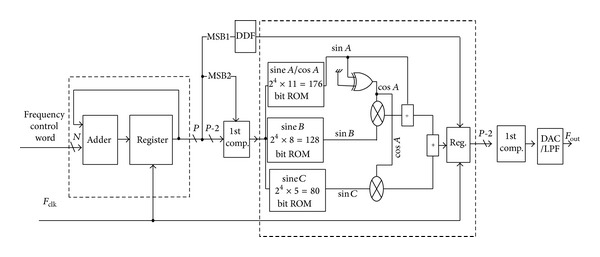
The block diagram of the final design of high-speed DDFS.

The final design of the high-speed DDFS, which consists of parallel pipelining PA and compressed ROM LUT by using the wave symmetry technique, is shown in Figure 7.

## 5. Hardware Implementation of High-Speed DDFS

The proposed design of the high-speed DDFS with 0.029 Hz frequency resolution was coded in Verilog HDL, successfully simulated in ALTERA Quartus II software, and implemented with a Cyclone III FPGA kit board.

**Figure 8:**
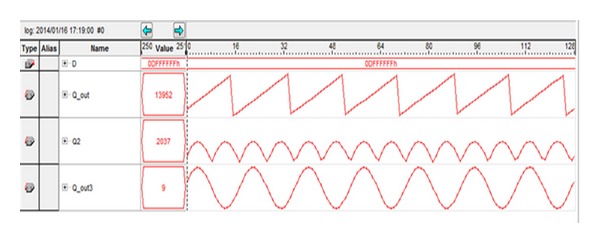
Signal tap logic analyzer of FCW input (in hexadecimal), the sawtooth PA output, and half and full amplitude waveform.

The proposed DDFS has been verified using a spectrum analyzer. The measured results are consistent with the simulated results. The DDFS design accumulates the FCW input into the sawtooth PA output, and the PA output feeds into the phase-to-amplitude converter (ROM LUT). The full amplitude waveform at the output of the ROM lookup table is shown using the Signal Tap logic analyzer in Figure 8.

## 6. Result and Discussion

**Figure 9:**
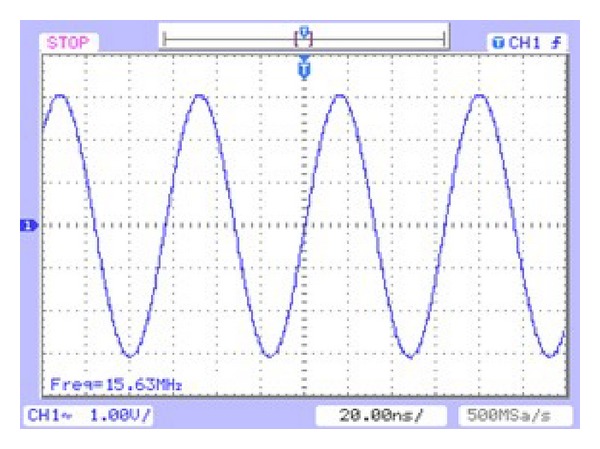
The sine wave signal of the high-speed DDFS.

The 32-bit DDFS has been implemented on the Cyclone III FPGA kit board. The 32-bit phase accumulator output was truncated into 14 bits. Approximately 196608 (2^14^ × 12) bits were required to draw the full sine wave signal with 12-bit output. Quarter-wave symmetry and an angular decomposition technique based on trigonometric identity were applied. The ROM was compressed to only 368 bits, with 534.2 : 1 ratio. The measured DDFS output waveforms and spectra for different clock frequencies illustrate the purity of the sine wave output as shown in Figure 9, for frequency tuning word = (1FFFFFFF) in hexadecimal format, and *F*_clk_ = 125 MHz (Cyclone III kit board clock frequency). The output frequency that was calculated is *F*_out_ = (FCW/2^*N*^) × *F*_clk_ = (2^29^ − 1/2^32^) × 125∗10^6^ = 15.624999 MHz. The measured output frequency from oscilloscope is 15.63 MHz and this is closely matched with the calculation result.

The signal-to-noise ratio (SNR) was approximated based on

(14) $$\begin{array}{c} \text{SNR}=-\;6.02M-1.76\;\text{dB} \end{array}$$

(see [29]), where *M* represents the number of bits used to feed the digital-to-analog converter. For the proposed DDFS, *M* = 12 and the calculated SNR is 74 dB.

**Figure 10:**
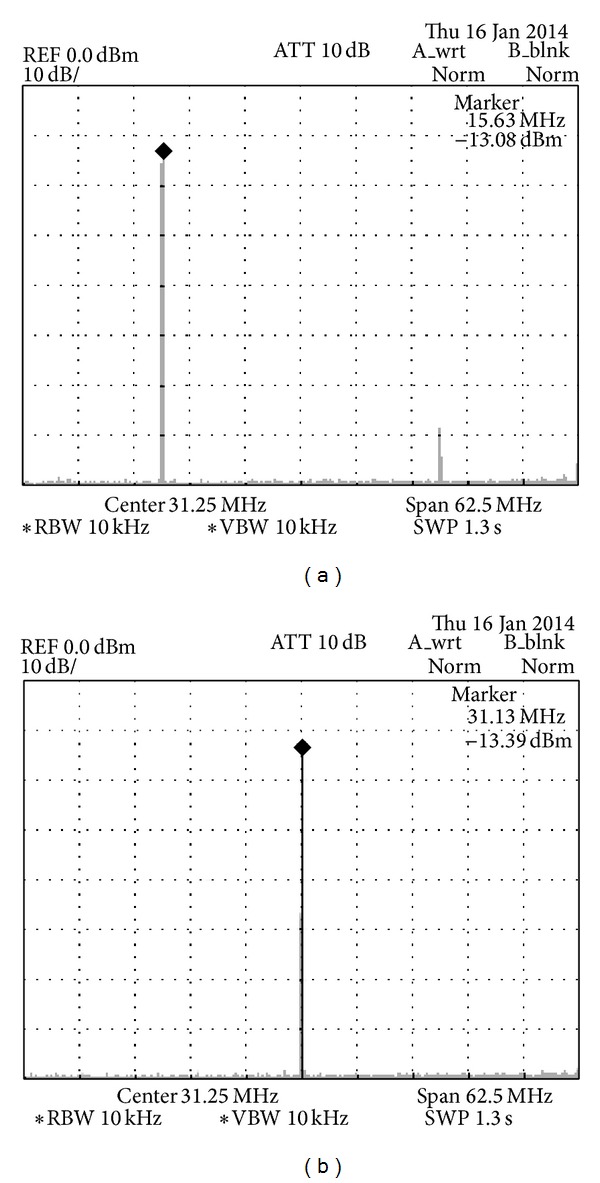
The measured signal-to-noise ratio for the DDFS waveform output (a and b).

The measured DDFS output from the spectrum analyzer shows SNR of approximately 68 dB as shown in Figures 10(a) and 10(b). The result is about 6 dB less than the calculated result and this is due to the noise from wire connections.

Table 1 shows the comparison of the ROM size used in the proposed design with some other parameters. The table shows that the proposed design has the smallest ROM size.

## 7. Conclusion

A 32-bit high-speed DDFS system was designed with 0.029 Hz frequency resolution. The system was successfully simulated in ALTERA Quartus II software. The phase accumulator together with the ROM lookup table has been implemented on the Cyclone III FPGA kit and verified with a Signal Tap logic analyzer, and the complete DDFS system with digital to analog convertor has been implemented on the Cyclone III FPGA kit board and the performance has been measured using oscilloscope and spectrum analyzer. Parallel pipelining with clock gating has been applied on a modified BK adder for the proposed phase accumulator design. Wave symmetry and an angular decomposition technique based on trigonometric identity were used to reduce the ROM size. The ROM was compressed to only 368 bits, with a 534.2 : 1 ratio. The proposed design has demonstrated attractive results that improve the operation speed and reduce the ROM size significantly.

## Figures and Tables

**Table 1:** Comparison between Rom size with previous DDFS works.

	Phase accumulator (bit)	Truncated phase (bit)	Amplitude phase (bit)	ROM size (bit)	Truncation ratio	SFDR (dB)
Sunderland et al., 1984 [11]	20	14	12	3328	59.1 : 1	72
Nicholas et al., 1988 [12]	31	15	14	3072	149.3 : 1	90
Curticǎpean and Niittylahti, 2001 [18]	28	14	12	832	236 : 1	84
De Caro and Strollo, 2005 [19]	24	14	12	480	409.6 : 1	83.6
Yang et al., 2004 [8]	32	14	12	2176	90.35 : 1	NA
Chimakurthy et al., 2006 [20]	15	15	15	1216	404 : 1	90.3
Babak and Keshavarzi, 2009 [21]	32	16	14	1664	551.3 : 1	85.3
De Caro et al., 2008 [22]	24	14	12	672	292.5 : 1	80
**This work **	**32**	**14**	**12**	**368**	**534.2 : 1**	**68**

Note: in this work the measured DDFS output waveform is in signal-to-noise ratio (SNR).
